# Teenage pregnancies resulting from rape in Sri Lanka – lessons learned

**DOI:** 10.1192/j.eurpsy.2021.1680

**Published:** 2021-08-13

**Authors:** Y. Rohanachandra

**Affiliations:** Department Of Psychiatry, University of Sri Jayewardenepura, Nugegoda, Sri Lanka

**Keywords:** teenage pregnancy, pregnancy following rape, pregnancy from sexual abuse, Sri Lanka

## Abstract

**Introduction:**

Rape resulting in pregnancy warrants special attention due to the associated psychosocial and physical adversities. There are no guidelines for the management of teenage pregnancies resulting from rape in Sri Lanka.

**Objectives:**

This case series aims to describe the experience of four teenagers who became pregnant as a result of rape in Sri Lanka.

**Methods:**

This is a case series of 4 pregnant teenagers who became pregnant as a result of rape

**Results:**

This case series highlight the deficiencies in services in Sri Lanka such as lack of legal framework to terminate pregnancy following rape, delay in legal procedure leading to prolonged institutionalization of pregnant teenager, not giving the teenage mothers the choice of breastfeeding and lack of awareness about the psychological consequences of rape and teenage pregnancy.

**Conclusions:**

Formulating a national guideline on managing rape related pregnancy in teenagers in Sri Lanka, with the involvement of all stakeholders is a need of the hour.

**Disclosure:**

No significant relationships.

